# Pathogenicity and virulence mechanisms of Lassa virus and its animal modeling, diagnostic, prophylactic, and therapeutic developments

**DOI:** 10.1080/21505594.2021.2000290

**Published:** 2021-12-21

**Authors:** Hannah L. Murphy, Hinh Ly

**Affiliations:** Department of Veterinary & Biomedical Sciences, Comparative & Molecular Biosciences Graduate Program, College of Veterinary Medicine, University of Minnesota, Twin Cities, MN, USA

**Keywords:** Lassa virus, arenavirus, mammarenavirus, pathogenicity, pathogenesis, virulence, vaccine, therapeutics, diagnostics, hemorrhagic fevers

## Abstract

Lassa fever (LF) is a deadly viral hemorrhagic disease that is endemic to West Africa. The causative agent of LF is Lassa virus (LASV), which causes approximately 300,000 infections and 5,000 deaths annually. There are currently no approved therapeutics or FDA-approved vaccines against LASV. The high genetic variability between LASV strains and immune evasion mediated by the virus complicate the development of effective therapeutics and vaccines. Here, we aim to provide a comprehensive review of the basic biology of LASV and its mechanisms of disease pathogenesis and virulence in various animal models, as well as an update on prospective vaccines, therapeutics, and diagnostics for LF. Until effective vaccines and/or therapeutics are available for use to prevent or treat LF, a better level of understanding of the basic biology of LASV, its natural genetic variations and immune evasion mechanisms as potential pathogenicity factors, and of the rodent reservoir-vector populations and their geographical distributions, is necessary for the development of accurate diagnostics and effective therapeutics and vaccines against this deadly human viral pathogen.

## Graphical Abstract

**Figure:**
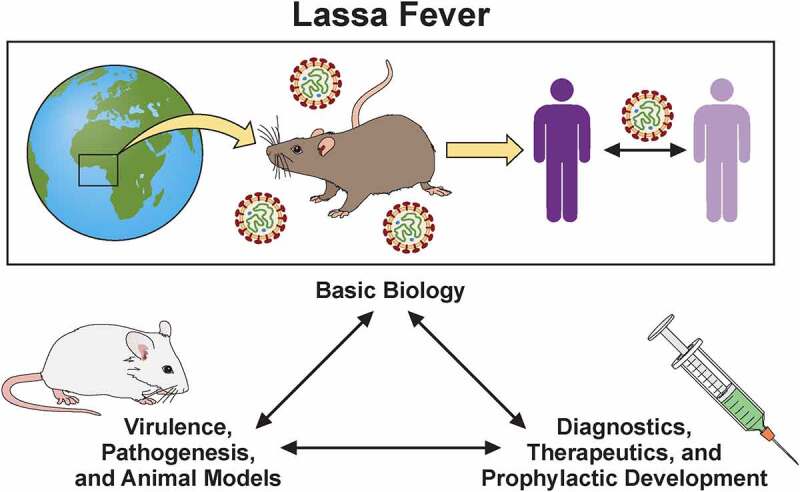

## Introduction

Lassa fever (LF) is an acute infectious disease in humans that is endemic in several countries in West Africa. The virus that causes the disease is called Lassa virus (LASV). It was first discovered in Lassa, Nigeria, in 1969 after two missionary nurses were fatally infected [1]. Although the disease is endemic to West Africa, travel-associated Lassa fever cases have been recorded in the USA, Europe, and Asia and were summarized in a recent review article [2]. Due to the high rate of infection and the fact that there are currently no approved vaccines or therapeutics against LASV, there is a concern that the virus could be used as a biological weapon [3]. According to the Centers for Disease Control and Prevention (CDC), an estimated 100,000–300,000 infections occur each year in West Africa with approximately 5,000 deaths annually; however, these numbers are probably underestimated because the methods used for estimations are relatively crude due to the lack of a standardized surveillance system for LASV, and its potential misdiagnoses for other infectious diseases, such as malaria, that are also endemic in this region [4,5]. LF has the second highest global burdens among all known viral hemorrhagic fevers, second only to Dengue fever which has an estimated 390 million infections per year, of which 96 million can manifest clinically [6–8]. Most LASV-infected individuals can mount an immune response strong enough to control the infection; however, some develop a severe form of LF which can culminate in death.

Based on population levels of people living in conditions that are suitable for zoonotic transmission of LASV, 37.7 million people are currently estimated to be at risk of contracting LASV in the African continent [9]. Despite a great number of people at risk of LASV infection, there are currently no FDA-approved vaccines or effective therapeutics against this form of an infectious disease. However, there are several vaccines and therapeutic candidates in various stages of preclinical development for LF. Several factors contribute to the lack of LF treatment modalities, including the relatively high genetic variability between LASV strains and immune evasion mediated by the virus. These factors present a real challenge for infected individuals to mount a robust immune response against the infection. For this reason, the World Health Organization (WHO) puts LF on their Blueprint list of priority diseases, which indicates the need for a greater understanding of LF pathogenicity and virulence to develop proper preventative, therapeutic, and diagnostic methods for LASV, as well as a standardized LF disease surveillance system. Therefore, the aim of this review is to provide a comprehensive review of the basic biology of LASV and of LF disease pathogenesis and pathogenicity, including the molecular mechanisms of LASV replication and immune evasion, as well as an update on prospective vaccines, therapeutics, and diagnostics for LF.

## LASV lineages and their geographical distributions

**Figure 1.**
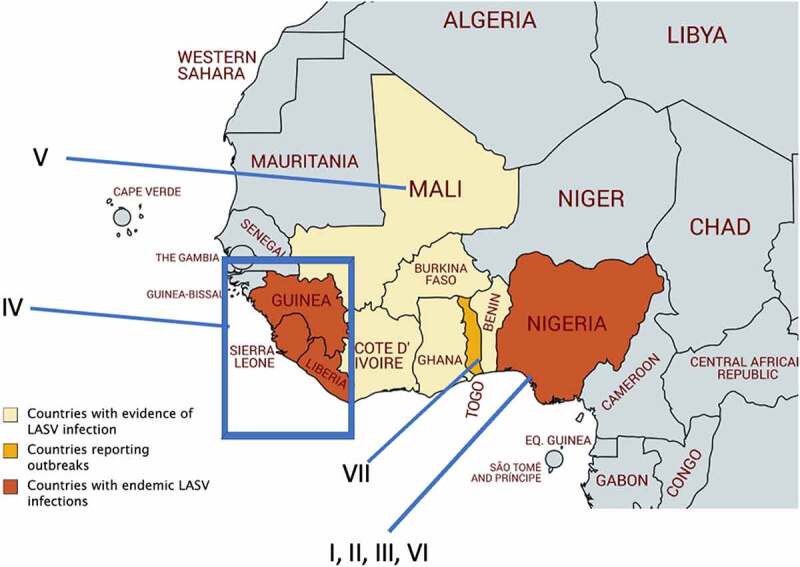
Geographical distribution of different Lassa virus (LASV) lineages in West Africa. LASV lineages are determined based on phylogenetic analysis of viral nucleotide or amino acid sequence variations and the geographical clustering and location where the viruses were discovered. Lineages I–III, and VI are circulating in Nigeria. The lineage IV is found in Guinea, Sierra Leone, and Liberia, and lineage V in southern Mali. The proposed lineage VII is found in Togo. Map made with an outline obtained from Africa – MapChart at the following web-site: https://mapchart.net/africa.html

LASV belongs to the virus family *Arenaviridae*, genera *Mammarenaviridae*, order *Bunyavirales*, and phylum *Negarnaviricota* [10–12]. Mammarenaviruses are further classified into two monophyletic groups organized by regions of virus origins, such as the Old World (OW) viruses and New World (NW) viruses. OW viruses include LASV and Lujo virus (LUJV) in Africa, and Lymphocytic Choriomeningitis virus (LCMV) which has a world-wide distribution. The NW viruses include Machupo virus (MACV), Junin virus (JUNV), Guanarito virus (GTOV), Sabia virus (SABV), and Chapare virus (CHPV) which are all in South America. There are four confirmed LASV lineages that are clustered into different West African regions (Figure 1). Lineages (I–IV) are firmly established and accepted by the scientific community and an additional three proposed lineages (V–VII) have been discovered in the last decade but are not yet fully established. Establishment of a new viral lineage requires phylogenetic analysis to determine geographical origin and the amount of sequence variation from preexisting lineages [13]. For example, LASV glycoprotein (GP) epitopes have diverging degrees of amino acid conservation within LASV lineages and this can be used as a determinant of variations. As complete LASV genome sequences become increasingly available, more complete phylogenetic comparisons can be made to determine the viral lineages.

LASV lineages are determined based on phylogenetic analysis of viral nucleotide sequence variations, geographical clustering, and the location where the viruses were discovered. Sequence diversity of the LASV genome is higher between lineages (up to ~25%) and more conserved within the same lineage [14]. Lineages I–III, and VI are circulating in Nigeria. Lineage IV is found in Guinea, Sierra Leone, and Liberia, and lineage V in southern Mali. The proposed lineage VII is found in Togo [13,15]. LASV sequence diversity is highest in Nigeria, and many of the sequences from human infections come from lineages II and IV [16]. It is noteworthy that LASV strain variation is significantly higher than Zaire ebolavirus, which has about 3% variation between strains [13]. LASV lineage diversity is mainly reflected in the sequence variations within the viral GP and nucleoprotein (NP) [13]. A mong the confirmed and proposed LASV lineages, the GP amino acid sequence’s inter-lineage variation is 4.9%-11% [13]. A comparison of full-length sequences of the LASV genome from each of the four lineages shows, in the NP gene, that there is a 23.8% nucleotide difference and a 12% amino acid difference, which is more variable than GP genes [17]. It is noteworthy that at the time when this study was done, the three new LASV lineages V, VI, and VII were not yet known. A significant amount of diversity in the LASV genes present a real challenge for developing an effective vaccine and suggests that natural viral gene variations may serve as a potential pathogenicity factor. For example, it has recently been shown that when cells transfected with an IFN-β promoter-directed luciferase plasmid and a plasmid expressing the activation domain of RIG-I or MDA5 [collectively known as RIG-I-like Receptor (RLR)] along with a Z protein expression plasmid, the natural sequence variations in the Z proteins of pathogenic mammarenaviruses [i.e., LASV, Dandenong virus (DANV), LCMV, LUJV, CHPV, MACV, GTOV, JUNV, and SABV] were found to confer the ability to strongly suppress -RLR-induced IFN-β promoter activity. Nonpathogenic mammarenaviruses, on the contrary, do not have this ability to suppress RLR-induced IFN-β promoter activity [18,19]. This study implicates a unique molecular mechanism of immune evasion mediated only by human pathogenic mammarenaviruses, the significance of which needs to be validated in a proper animal model, such as a non-human primate model that will be discussed in a later section.

## LASV genome structure, replication strategies, and general life cycle

**Figure 2.**
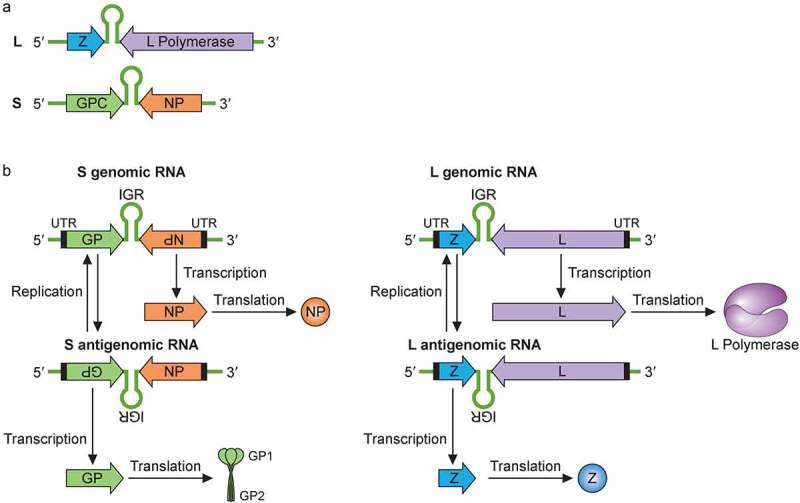
Mammarenaviral RNA genome structure, replication, transcription and gene expression strategies. (a) The bisegmented genome of arenaviruses (e.g., LASV) contains two genomic RNA segments that code for four known viral proteins in an ambisense coding strategy. Each genome segment contains two open reading frames that encode two gene products separated by a noncoding intergenic region (IGR) that forms stable hairpin RNA structure(s). The L segment is ~7.2 kb and codes for the L protein and the Z protein, while the S segment is ~3.5 kb and codes for the viral glycoprotein precursor (GPC) and NP proteins. (b) Mammarenaviral RNA replication, transcription and gene expression strategies. The L polymerase, together with NP, transcribes negative-sense genes (NP and L genes) starting at the 3ʹ untranslated region (UTR) toward the noncoding intergenic region (IGR) of the S genomic and L genomic RNAs in order to generate the viral L and NP mRNAs, respectively, from which the viral NP and L proteins are translated. Occasionally, the L polymerase, together with NP, continues RNA synthesis past the IGR to generate complementary S antigenomic RNA and L antigenomic RNA, which are used as templates to transcribe the GPC and Z mRNAs for translation into the respective viral proteins GPC and Z. GPC is post-translationally modified and processed by cellular proteases into GP1, GP2 and SSP protein subunits, which are incorporated into the cellular surface membrane where they interact with the viral Z protein and the viral ribonucleoprotein complex that consists of the viral NP, L and viral genomic RNAs for viral assembly and budding. The S and L antigenomic RNAs are used as templates by the viral L polymerase and NP to synthesize the full-length S and L genomic RNAs for incorporation into the viral RNPs for packaging into the newly formed virion particle

LASV’s genome is single-stranded, negative-sense RNA that is bisegmented. The large (L) and a small (S) segment encode four viral proteins in an ambisense coding strategy (Figure 2a) [20]. Each genome segment contains two open reading frames that encode two gene products separated by a noncoding intergenic region (IGR) that forms stable hairpin RNA structures. The IGR primary sequences and secondary structures are thought to help terminate viral mRNA transcription [20]. The L segment is ~7.2 kb and codes for the L protein and the Z protein, while the S segment is ~3.5 kb and codes for the viral glycoprotein precursor (GPC) and NP proteins. The L protein is an RNA-dependent RNA polymerase (RdRP) that, together with the viral NP, mediates viral RNA transcription and replication. The Z protein is a zinc-finger motif containing matrix protein, which carries out multiple functions during the viral life cycle, including regulating viral RNA synthesis, orchestration of viral assembly and budding via its interactions with some cellular proteins at the cell surface membrane, and antagonizing the host type 1 interferon (IFN-I) system [18,19,21–24]. Besides L, Z and GPC, the genome of mammarenaviruses (also known simply as arenaviruses), such as LASV, enccodes the NP that encapsulates the bisegmented viral genome and serves as the main structural component of the mammarenaviral ribonucleoprotein (RNP) complex. NP also functions to support viral replication and has been shown to modulate the host immune response by degrading the virus-associated double-stranded RNAs (dsRNAs) via the NP exoribonuclease function [25,26].

The life cycle of all known mammarenaviruses, including that of LASV, starts with the viral GP-mediated cellular entry. All known OW mammarenaviruses (e.g., LASV, LCMV) and clade C of the NW mammarenaviruses use α-dystroglycan as the main cellular entry receptor [27]. Receptor binding is mediated by the interaction of the LASV GP1 C terminal domain with α-dystroglycan, which allows viral particle internalization and delivery into the late endosomes [28]. Acidification inside of the endosome causes LASV to change its binding affinity of α-dystroglycan for an endosomal receptor known as the lysosomal-associated membrane protein (LAMP1) [29]. It has been proposed that LAMP1 increases efficiency of LASV entry and infection by elevating the pH threshold for GPC-mediated fusion, thus avoiding virion inactivation by low pH and proteases, which are usually present in LAMP1-negative endosomes [30]. The change in pH allows the release of the viral genome as an RNP complex into the cellular cytoplasm. Once the RNP is delivered into the cytoplasm, viral RNA synthesis ensues.

With the ambisense coding strategy, NP and L genes are coded in a negative-sense orientation on the viral genomic S and L segments, respectively (Figure 2b). The viral L polymerase, together with NP, starts to transcribe L and NP genes into mRNAs in a process that involves cap snatching [31]. Because mammarenaviruses lack the machinery to make their m^7^G 5ʹ cap structures, they must steal (or “snatch”) these cap structures from cellular mRNAs for use as primers to initiate transcription. Cap snatching is mediated by the endonuclease activity located at the N-terminus of the L polymerase [32]. While mammarenaviral mRNA transcripts have 5ʹ caps, they are not poly-adenylated at their 3ʹ ends [33]. Instead, viral transcription is terminated when the L-NP polymerase complex reaches the IGRs that form thermodynamically stable RNA stem-loop structure(s), which are thought to protect the viral mRNAs from being attacked and degraded by host ribonucleases (RNases). Occasionally, however, the viral L-NP polymerase complex can start *de novo* RNA synthesis at the viral 3ʹ distal ends (without the use of a cap structure) and can suppress the early terminating signal of the IGRs to fully copy the viral genomic RNAs (gRNAs) into complementary RNAs as full-length agRNAs (Figure 2b).

NP is the most abundantly expressed protein and participates in viral genome replication and transcription [34]. Using an LCMV minigenome assay that expresses the chloramphenicol acetyltransferase (CAT) reporter gene upon the expression of viral L and NP proteins from cloned cDNAs, Pinschewer and colleagues showed that replication and transcription of the LCMV RNA were enhanced by increasing levels of intracellular NP. Low levels of NP at the beginning of the virus infection cycle appear to prevent the viral NP-L polymerase complex from reading through the IGR, therefore favoring viral transcription over replication. However, as NP accumulates during the virus infection cycle, the viral polymerase shifts to a viral RNA replication mode and read through the IGR to generate a full-length agRNAs, which will serve as templates for the synthesis of the GPC and Z mRNAs [34]. Since the GPC and Z genes are coded in the positive orientation on the genomic S and L RNA segments, respectively (Figure 2b), they must first go through a round of full-length viral RNA replication to generate the agRNAs before these genes can be transcribed into mRNAs [35]. The viral L-NP polymerase complex can also use agRNAs as templates to newly synthesize full-length genomic RNAs (gRNAs) for packaging into the virion particles during the virion assembly process, which involves all components of the viral proteins and gRNAs that are thought to coalesce at the cell surface membrane. However, before viral assembly process can occur, the viral GPC must first be synthesized and modified within the proper cellular compartments.

The GPC gene located on the S segment of the viral genome must first be replicated before being transcribed and translated into the glycoprotein precursor (GPC) polypeptide (Figure 2b). The GPC polypeptide that consists of the GP1, GP2, and the Stable Signal Peptide (SSP) sequences, is then sequentially cleaved by the host cellular signal peptidase (SPase) and subtilisin kexin isoenzyme-1/site 1 protease (SK1-S1P) and into three subunits SSP, GP1, and GP2 [36–39,40]. The three subunits of the GP2 form a trimeric protein complex that is anchored on the surface membrane of the host cell, where it interacts with the GP1 subunit and with the SSP subunit that spans the cellular membrane and, ultimately, the virion particle, after it buds off the cells during the virion egress process [41]. GP1 is exposed on the surface of the virion particle where it interacts with the main host entry receptor (e.g., α-dystroglycan for all known OW viruses and clade C NW mammarenaviruses and Transferrin Receptor 1 (TfR1) for clade B of NW mammarenaviruses), while GP2 mediates viral fusion via a structure and mechanism resembling class I viral fusion proteins [42,43].

The SSP is implicated in fusion, proper transport, and maturation of the viral GP. It is noteworthy that unlike other known viral GPs, mammarenaviral SSP is unusually long (i.e., approximately 58 amino acids) [38], whereas other conventional viral SSPs consist of 18–30 amino acids [40]. Unlike other conventional viral SSPs that are not part of the final glycoprotein complex, mammarenaviral SSP remains an essential part of the mature GP complex on the surface of the virion to help mediate the membrane fusion process during virus entry into cells [44,45]. Ordinarily, conventional viral and cellular SSPs are comprised of three distinct regions: an “h-region” that consists of a long stretch of hydrophobic amino acids that tends to form a single alpha-helix, a polar N-terminal region of variable length, and a C-terminal region that contains the signal peptidase cleavage site [46]. In eukaryotic cells, SSPs target nascent secretory and membrane proteins to the endoplasmic reticulum (ER) and mediate insertion of polypeptides into the translocon [46]. SSPs are thought to be incorporated into the cellular membrane in a loop-like manner which ultimately results in the N terminus exposed into the cytoplasm; the SSP is then cleaved by SPase on the luminal side of the membrane [47]. Unlike conventional SSPs, mammarenaviral SSP has two conserved hydrophobic domains that are separated by a lysine residue [48]. Mammarenaviral GPC is translated in the ER where SSP is cleaved from GP1/GP2 by SPase [37]. Additionally, since the SSP of mammarenaviruses is an integral component of the mature GP that is incorporated into the cell surface membrane and subsequently virion membrane, it has an extended lifespan as compared to other viral and cellular SSPs [48].

The GPC mRNA is translated in the endoplasmic reticulum (ER) into the glycoprotein precursor polypeptide that is first cleaved by the cellular SPase into SSP and the GP1/GP2 polypeptide, which is further cleaved in the Golgi apparatus by the cellular SKI1/S1P into GP1 and GP2 subunits that are further modified by glycosylation [36–40]. The three viral glycoprotein cleavage products form a glycoprotein spike trimer on the cellular surface membrane. Virus budding is then mediated by a combination of some cellular and viral proteins [for a recent review on mammarenavirus assembly and budding, see [43,49]]. Briefly, it was determined that the C-terminal domains (known as the Late domain) of the viral Z protein are critical in viral assembly, budding, and release of new virions [50]. The Z protein’s C-terminus interacts with L, NP, GP, and the cellular endosomal sorting complexes required for transport (ESCRT) proteins to ensure the co-localization of all viral proteins as well as the viral genomic RNAs for proper ribonucleoprotein complex (RNP) assembly [50]. The Z protein then uses its N-terminal myristoylation site to anchor into the plasma membrane. Z protein then regulates virus-host protein-protein interactions to form and release newly formed virion particles into the extracellular milieu [23].

## Lassa disease pathogenesis

### LASV transmission and case fatality rates

The majority of LF is caused by rodent-to-human LASV transmissions from direct contacts with infected animals or animal excreta, but there have also been reports of human-to-human transmissions (see Graphical Abstract) [51]. It is estimated that 20% of LF cases could be due to human-to-human transmissions with many cases caused by the so-called “super-spreaders” [52]. Human-to-human transmission happens most often as a nosocomial infection where personal protective equipment (PPE) is not readily available or is not being used properly, from contaminated medical equipment, or hospital/laboratory accidents from needlesticks [53]. Inhalation of infected rodent excreta is thought to be the most common form of LASV transmissions since rodents readily live inside and around homes. Butchering and eating infected rodent meat is another potential route of exposure as bushmeat is sometimes used as an important food source in West Africa [5]. Although LASV infection can cause symptomatic disease in humans, it is believed to cause asymptomatic infection in its natural animal reservoirs, such as the multimammate rodent species known as *Mastomys natalensis, Mastomys erythroleucus, Hylomyscus pamfi*, and in experimentally infected rodents, such as *Mus musculus* (house mouse), *Rattus rattus, Rattus fuscipus, Rattus fuscipus*, and *Myosoricinae soricidae* (shrew) [54,55]. This suggests a long-standing coevolution between LASV and the wild rodent species that can serve as reservoirs for the virus [53].

LF cases in West Africa were found to be highest in the dry season from January to March and lowest in the wet season from May to November; the seasonality of LF cases appeared to correlate with the seasonal reproductive cycle of the multimammate rat reservoir hosts which starts after the rainy season and extends well into the dry season [56,57]. People of all ages are susceptible to LASV regardless of the season. The current prevalence of LF is unknown as all available estimates are based on studies in the 1980’s when methods for clinical diagnostic and surveillance system for LASV were relatively primitive. To get an accurate figure on LF disease prevalence, the Coalition for Epidemic Preparedness Innovations (CEPI) is currently studying the prevalence of LF in Nigeria and some of its neighboring endemic countries in West Africa. According to CEPI, due to the significant variability and severity in LF disease symptoms and a dirt of formal and standard diagnoses, the true number of LF cases is likely much higher than the current estimate of 100,000 cases per year [58].

Based on statistics taken from rural populations by the United National Development Programme and the epidemiology of LF, there are currently an estimated 59 million seronegative people at risk of LASV infection based on the number of seroconversions per year, the number of annual infections, and the annual ratio of fatalities due to LASV infection [57]. The estimated case fatality rate (CFR) in the general population is 1% to 2%, which is much lower than the fatality rate in hospitalized patients [59]. The higher hospital CFR is based on the ability to correctly diagnose LF in the hospital setting and the fact that people are generally already quite ill when they were admitted into the hospital. In endemic areas, the CFR in hospitalized LF patients is between 9.3% to 18%, but nosocomial or community-based outbreaks of LASV have a higher CFR at 36% to 65%, perhaps due to the inadequate health care infrastructure in endemic communities [4]. In the most recent 2018–2019 Nigerian LF outbreak, the case fatality rate was as high as 25.4% [60]. The CFR of LF in children (at 12%-14%) is generally higher than that of adults [61,62]. The increased CFR in children has been hypothesized to be attributed to children spending more time in and around the home and thereby increasing their chances of exposure to LASV-infected rodents. Since women of all ages have a higher proportion of LF than men, perhaps because women in poor or rural communities are more likely to be homemakers that increase their chances of LASV exposure from infected rodents [63,64].

### LF disease signs and symptoms

After exposure, the incubation period for LF is approximately 7–21 days. According to the WHO 80% of individuals infected with LASV are asymptomatic, whereas 20% of infected individuals experience severe and multisystem disease [WHO, 64]. Early signs and symptoms of LF are generally mild and similar to other febrile illnesses, which makes LF clinical diagnosis difficult. West African patients, or patients who have recently visited the known LF endemic area, with a fever above 38°C (100.4 °F) and are not responding to antimalarial or antibiotic treatments should be suspected to have LF [57]. Individuals with mild LF disease usually experience influenza-like symptoms that include fever, weakness, malaise, and headaches [62].

As the disease progresses, joint pain, lower back pain, nonproductive cough, and sore throat usually ensue during the early phase of the disease. Seventy percent (70%) of severe LF patients have pharyngitis with yellow to white exudate patches that can appear on the tonsils in a pseudomembrane [65]. Fifty to seventy percent (50%-70%) of symptomatic LF patients have diarrhea and experience vomiting and abdominal discomfort [62]. Recovery from mild disease usually occurs 8–10 days after symptoms onset. However, severe LF cases rapidly deteriorate 6–10 days after viral infection. Certain types of symptoms and viremia levels can be predictive of the disease outcome. Patients with serum viral titers higher than 10^3^ TCID_50_/mL (TCID_50_ is determined by the concentration of virus that infects 50% of the target cells in a mammalian cell culture) and high levels of aspartate aminotransferase (AST, a cellular enzyme indicative of tissue damage) are 21 times more likely to have a fatal outcome [66]. Viremia level peaks 4–9 days after the onset of symptoms, but it subsides as the virus is cleared from the blood usually at three weeks after symptoms onset. However, individuals with severe LF symptoms and high viremia levels often fail to elicit a proper immune response to control virus dissemination, which ultimately results in a poor prognosis or death.

Increased vascular permeability, resulting in facial edema and pleural and pericardial effusions, is also common in severe LF cases. Severe LF patients can also experience acute respiratory distress with laryngeal edema and fluid accumulation in the lung cavity [62]. In 15%-20% of severe LF cases, mucosal bleeding is present with low blood pressure [62]. Death usually occurs within 14 days following hypovolemic shock and signs of encephalopathy. Disorientation, gait anomalies, convulsions, comas, and seizures are also possible in the later stages of the disease, with tremors seen a few hours before death in some patients [62].

### LF disease pathogenesis in pregnant women

The signs and symptoms of LF in pregnant women are usually nonspecific and indistinguishable from other febrile illnesses during the early phase of the LASV infection [67]. However, LF is severe in pregnant women, especially when they are infected late in the pregnancy, when vaginal bleeding and spontaneous abortion can occur. High rates of fetal and maternal mortality correlate with high viral loads in the maternal blood, placenta, and fetal tissues [68,69]. Fetuses are spontaneously aborted from infected mothers at the rate of 92% in early pregnancy and 75% in the third trimester of gestation [68,70,71]. The rate of maternal mortality is around 7% if the mother is infected in the first two trimesters of pregnancy, which can increase up to 30% if she is infected during the third trimester, and 50% if she is infected within one month of delivery [70,71]. It has been reported that pregnant women were almost three times as likely to die from LF than their non-pregnant counterparts [67].

There have been reports of LF cases with good maternal outcomes, but it is rare for fetuses to survive LASV infection [72,73]. Usually, a significant correlate for a poor outcome of the mother and the fetus is a diagnosis of LF in the third trimester. However, [74] did not observe this effect in their retrospective study of pregnant mothers in a south Nigerian hospital [74]. It is noteworthy that this hospital used the antiviral ribavirin as an early therapy for all LF cases, unlike other Nigerian hospitals which used uterine evacuation and deferred ribavirin in pregnant women. Deferment was chosen because ribavirin has been shown to be teratogenic in animal studies and that there is a lack of testing of the safety of ribavirin in pregnant women.

### LF pathogenesis in children

LF is not well studied in children, partly because diagnosing it in this age group of patients is more challenging than that in adult patients. The LF disease manifestations are generally mild, and the symptoms are mostly nonspecific in younger patients. Typical LF symptoms in adult and older pediatric cases include sore throat, retrosternal pain, and malaise that are hard to judge in neonates as these symptoms are common in many diseases [61,62]. LF symptoms in neonates may include a high-grade fever, swollen lymph nodes, convulsions, and hemorrhaging that can commonly be misdiagnosed as neonatal sepsis [75,76]. Neonates, infants, and toddlers can also experience a severe form of LF known as swollen baby syndrome that is characterized by widespread edema, abdominal distention, and hemorrhaging [61,75,77]. In older children, LF symptoms can manifest as diarrheal disease, pneumonia, prolonged fever, or those which are more typical of adult LF symptoms [61,62,76,77]. Like adults, hemorrhaging, acute renal failure, convulsions, and comas are indicators of poor outcomes in children infected with LASV [78].

## Lassa disease pathology

### Gross and microscopic pathological changes

The gross pathological study of LF in humans (i.e., autopsy) has been limited by several factors, including civil unrests, the underdeveloped biomedical infrastructure in West Africa, and regional customs of not violating corpses [71,79]. As a result, our understanding of LF pathology is based on limited human data and by carefully extrapolating data from non-human primate (NHP) models of LF [71]. Evidence collected from autopsies of deceased LASV patients and from experimentally infected NHPs shows a lack of vascular lesions, which correlates with the lack of cytopathic effect seen with LASV infection. However, there is evidence of increased permeability of the vascular endothelium, the mechanism of which is unknown [80]. It is possible that vascular endothelial permeability is a result of viral infection of the endothelial cells which causes cellular changes to allow increased fluid accumulation and edema in some severe LF cases [80].

There is clinical evidence of lesions in the spleen, liver, and adrenal glands of some LF patients. Additionally, pleural and pericardial effusions, pulmonary edema, ascites, and hemorrhagic manifestations of the gastrointestinal system are common [1,71]. Postmortem microscopic pathological changes, such as necrosis of hepatocytes, splenocytes, and adrenocortical cells, are also common [1,80,81].

The most common liver lesions include focal cytoplasmic degeneration of hepatocytes, multifocal hepatocellular necrosis, monocytic reaction to necrosis, and hepatocellular mitosis [81], which usually coincide, but have variable levels of severity. Spleen samples show evidence of eosinophilic necrosis and lymphoid depletion, atrophy of white pulp, and infiltration of lymphocytes and mononuclear cells [80]. Splenic necrosis has been found in the marginal zone of the periarteriolar lymphocytic sheath, and fibrin has been found among the necrotic cellular debris [81].

### LASV-induced sensorineural hearing loss

Neurological sequelae are common in LF survivors and can include memory loss, ataxia, neuromuscular pain, and sensorineural hearing loss (SNHL). SNHL occurs in about one-third of LF survivors and can develop bilaterally or unilaterally. In two-thirds of those cases, hearing loss is irreversible, but it is hard to know exact numbers since follow-up patient data are not always available [3,59,82]. SNHL prevalence in LF endemic countries is likely underestimated, and as such, LF-associated SNHL is considered a neglected public health and social burden. Quality of life can be severely impacted for individuals with LASV-induced SNHL, as they are more likely to be unemployed (due to tinnitus and vertigo) that can restrict their economic and/or social status [59,83].

SNHL is characterized by damage to the cochlear hair cells and a hearing loss of 30 dB or greater where over three different auditory frequencies are tested [84]. It usually develops in the late stages of an acute phase or early during the convalescence phase of the LF disease [85]. Despite a clear correlation between SNHL and LF infection, the underlying mechanisms for hearing loss have not yet been fully understood. Treatment of LF patients with ribavirin was thought to contribute to hearing loss; however, recent studies found no association between hearing loss and ribavirin administration [59,86,87,88]. There are currently three suggested mechanisms of LASV infection-mediated SNHL, which include immune-mediated mechanism, direct viral damage to the inner ear, or a combination of both [88]. Some studies suggest that LASV-induced SNHL is either entirely or at least partially due to an immune-mediated process [89,90]. A nonlethal LASV infection model of immunodeficient STAT1 knock-out (KO) mice exhibits permanent SNHL and damage to inner hair cells and the auditory nerve [89]. T cells were also found in the damaged spiral ganglion of these LASV-infected immunodeficient mice. It is noteworthy that the authors of this study measured the behavior, (i.e., how far mice moved in response to sound), instead of electrical signaling as a measurement of the degrees of hearing loss.

LASV-induced SNHL studies in NHPs (i.e., cynomolgus macaques) showed evidence of an immune-mediated vasculitis-like syndrome that might involve the inner ear as a potential pathological mechanism of hearing impairment [90]. Gross pathological findings showed severe lesions in several organs like those found in human’s autoimmune-associated vasculitis. Prior to euthanasia, LASV-infected NHPs showed elevated levels of autoimmune-associated serological markers of vasculitis and satisfied seven out of ten known criteria of autoimmune-associated systemic vasculitis in humans [90]. Interestingly, NHP tissue samples of the inner ear adjacent to the cochlear nerve showed moderate subacute to chronic-active perivascular inflammation in surrounded branches of the cochlear nerve, suggestive of an immune-mediated inflammatory response to LASV. However, a major limitation of this study is that the authors did not characterize the cochlear itself.

While some studies point toward an immune-mediated mechanism of LASV-induced SNHL [59,89,90], other studies suggest a direct damage of the tissues by virus infection as a potential cause of some cases of LF-associated SNHL [85]. In a case-control study of LF patients with SNHL in Nigeria, 40% of SNHL patients lacked antibodies against LASV which suggest that the antigen-antibody immunological reaction might not be responsible for all cases of LASV-induced SNHL, although one could argue that a patient could be clinically ill with LF without having detectable levels of circulating antibodies against LASV or that the serological survey was done early before any detectable antibody developments. The authors of this study also argued that if LASV-induced SNHL was immunologically induced, it would be expected for all hearing loss cases to be bilateral. However, unilateral SNHL associated with LF has been documented [85]. The fact that some cases of SNHL in LF are unilateral suggests that some SNHL can be caused by a direct virus-mediated damage to the auditory system. For example, rubella virus causes both bilateral and unilateral SNHL. Yet, in many of the cases, it is unilateral hearing loss that has been associated with a direct viral damage to the cochlear, specifically cell death in the organ of Corti and the stria vascularis [91,92]. Ibekwe and colleagues have also postulated LASV’s direct invasion of the hearing pathway as a possible mechanism of LF-associated SNHL [3]. Overall, the mechanisms of LASV-induced SNHL are still not yet fully understood and therefore warrant further investigations in an ideally immunocompetent small animal model.

## Molecular basis of interplays between LASV and the immune system

LF is characterized by a generalized immune suppression caused by inhibition of innate and adaptive immune responses to viral infection. Macrophages and dendritic cells (DCs) are innate immune cells that are targeted early in LASV infection with macrophages as the main cellular targets driving viral spread as these virus-infected cells are filtering into the draining lymph nodes and back into the tissues and organs [93–96]. Interestingly, macrophages and DCs are not activated upon LASV infection as evidenced by the lack of activation markers and cytokine expressions by these cells (e.g., CD80, CD86, CD40, TNFα, IL1β, IL6, and IL12) [93,96,97]. LASV-infected DCs and macrophages also fail to mature, resulting in the failure to secrete proinflammatory cytokines, leading to the lack of costimulatory molecule stimulation that is necessary for the proliferation of T cells or memory recalls of the adaptive immune cells to the pathogen and its immunogenic antigens [95]. For example, when LASV antigens are presented to T cells by immature DCs *in vitro*, they can induce a condition of T cell tolerance rather than activation, which can blunt T cell responses and therefore dampen the adaptive immune response to LASV infection [97,98]. In contrast, effective CD4 and CD8 T cell-mediated responses, but not B cell-mediated response, early during LASV infection in humans are critical for recovery from LF disease [99,100]. Patients who recover from acute LASV infection do so without a measurable neutralizing antibody response, and antibodies only develop in low titers late during the convalescence phase [101–103].

Vaccines that can induce a robust level of T cell responses, but not antibody responses, against LASV glycoproteins have been found to be protective against LASV infection in NHPs and guinea pigs, which suggest that antibodies may be less important for protection against LASV infection in these animals [104]. However, LASV infection does induce IgM and IgG productions, but their titers do not appear to correspond with clinical outcomes [66,105]. LF patients and experimentally LASV-infected animals who displayed high levels of circulating activated T cells and chemokines (e.g., IL-8 and CXCL-10 that are involved in T cell recruitment and activation) could recover from the infection. In contrast, individuals with low chemokine and T cell levels and delayed immune cellular activation often succumbed to the disease [102].

It would be reasonable to assume from the correlation of delayed T cell responses and fatal infections that T cells would be entirely beneficial for protecting against LASV infection; however, T cells have been suggested to play a dual role during LASV infection. T cells can help clear the infection, but in cases when virus clearance is unsuccessful, they can become a facilitator of disease pathogenesis, and as such, they can serve as a liability or pathogenicity factor. Flatz and colleagues have used a major histocompatibility complex-1 (MHC-I) mouse model to show that bystander T-cells can be generated after LASV infection, and that these cells migrate to the inflamed tissues and can cause tissue damage [106]. Tissue damage in inflamed tissues shows evidence of nonspecific T-cell activation [107,108]. There is evidence of correlation between T-cells that are antigenically unrelated to LASV and the hyper-expansion of T-cell clones in early time points after the onset of severe disease. There is also speculation that fatal outcomes of LF may be due to nonspecific T-cell clones dominating the immune response, which can dampen their ability to control virus replication [108,109].

The dual role of T cells in LF disease pathogenesis has also been studied in a humanized MHC-I and T-cell deficient mouse models. Unlike wild-type mice, these humanized mice are susceptible to LASV infection and are unable to control virus replication [106]. Humanized MHC-I mice that were depleted of T cells did not develop disease despite having high viremia, thus suggesting that T cells are more essential for rapid virus clearance than disease pathogenesis. The authors also postulated that the lack of extensive DC and macrophage activations in the T cell-depleted mice indicates that T cell responses contribute to the deleterious innate inflammatory reaction and hence LF disease pathogenesis.

**Figure 3.**
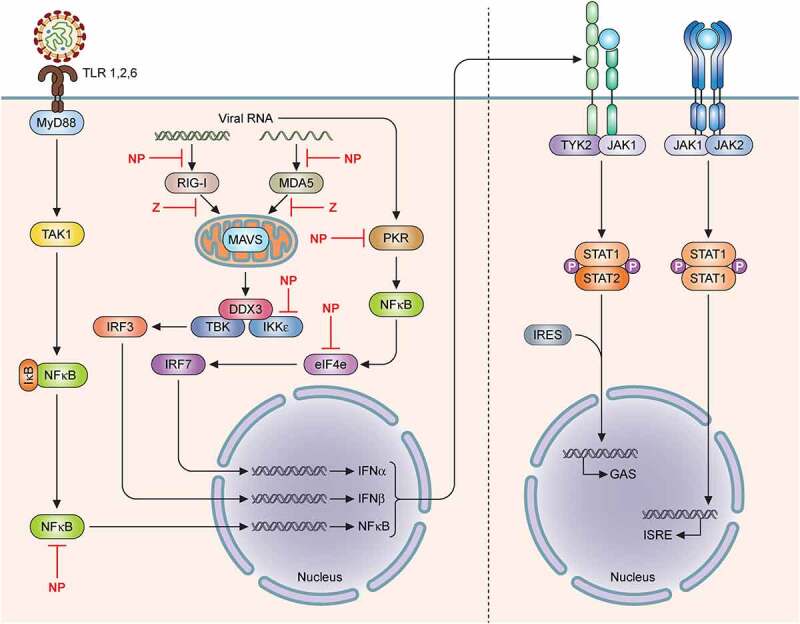
Mammrenavirus inhibition of innate immunity. The cellular Toll-like receptors (TLRs) recognize pathogen-associated molecular patterns (PAMPS) (e.g., viral glycoproteins) and utilize the cellular MYD88 adaptor protein to transmit a cellular signal to successively influence the activities of other cellular proteins, including TAK1, IKKα, in order to activate transcription factors, such as interferon regulatory factors (IRF3 and IRF7) and NFkB subunits to induce the expression of type I interferons (IFNα and IFNβ), which are secreted out of the cells to bind to their respective receptors on the surface membrane of the cells to further activate cellular protein kinases (TYK2, JAK1, JAK2) and transcription factors (STAT1 and STAT2 by phosphorylation and IRF9), which are translocated from the cellular cytoplasm into the nucleus to activate the expression of hundreds of antiviral genes from their respective promoters (GAS or ISRE). In addition to the TLRs, other intracellular receptors (e.g., RIG-I and MDA5) can recognize aberrant viral double-stranded RNAs (dsRNAs) as PAMPs to activate other cellular proteins (MAVS on the mitochrondria, DDX3, TBK, IKKe), which in turn, activate IRF3, IRF7 and NFkB transcription factors in order to upregulate the expression of type I interferon genes (IFNα and IFNβ). Aberrant virus-associated dsRNAs can also act as PAMPs to activate other intracellular receptor (PKR) to activate NFkB and influence the activity of the other cellular protein (e.g., eIF4E) in order to increase the expression of the type I interferon genes (IFNα and IFNβ). Mammarenaviral proteins, such as LASV NP and Z proteins, have been shown to use various strategies to inhibit the expression of type I interferon genes (IFNα and IFNβ)

Another mechanism that LASV uses during infection to suppress the immune system includes inhibition of the type 1 interferon (IFN-I) system that involves mainly IFNα and IFNβ expressions (Figure 3). Activation of the IFN-I system is essential in coordinating the subsequent cellular and adaptive immune responses to control and clear the infection. Experimentally infected mice that are deficient in the IFN-I pathway succumb to LASV infection, and IFN-I expression is suppressed in patients with severe LF [103,110]. In experimentally infected NHPs, IFNα expression is upregulated early during LASV infection, but in those that succumb to the infection, IFNα expression is strictly suppressed and is only upregulated again at late time points right before death, the reasons for and mechanisms of which are unclear [99].

The Z and NP proteins of LASV have been implicated to specifically inhibit IFN-I expressions [for a recent review, see 111]. The C-terminal exoribonuclease domain of NP has been shown to bind to IKKɛ and prevent the activation of downstream transcription factors, IRF3 and IRF7, which are required to induce the transcription of IFN-I genes (Figure 3) [112–114]. Mutagenesis studies of the LASV NP C-terminal 3ʹ-to-5ʹ exoribonuclease domain have identified DEDDH residues that are required to mediate increased levels of IFN-I in DCs and macrophages [25,26,115]. These amino-acid resides constitute the LASV NP’s exoribonuclease domain that specifically degrades dsRNAs that are aberrantly produced and therefore recognized by the infected cells as the pathogen-associated molecular patterns (PAMPs) to mediate IFN-I expression . This appears to be a unique viral NP enzymatic function that is not related to its role in viral RNA replication and transcription but serves to dampen the essential functions of the RIG-I-like receptors (e.g., RIG-I and MDA5), which are specifically used by cells to recognize PAMPs to confer resistance to viral infection [110, 116–118].

As the cellular protein called protein kinase R (PKR) can also be activated by dsRNAs (PAMPs) upon viral infection to enhance IFN-I production [119], some studies have investigated whether the molecular pathway of IFN-I production via PKR can be triggered by LASV infection [128,121]. Interestingly, it was found that whereas the NW mammarenaviruses (JUNV and MACV) can trigger PKR activation, the OW LASV cannot. It was thus postulated that LASV-associated dsRNAs are not recognized by PKR and therefore LASV can evade recognition by PKR through a mechanism that is yet to be clarified [120]. The lack of PKR activation by LASV can partly explain how this virus can dampen the innate immunity system as well as the cellular inflammatory response pathway. A possible mechanism for PKR evasion by LASV could be through a PKR-interacting protein known as the DEAD-box ATP-dependent RNA helicase (DDX3). Recent proteomics studies have identified DDX protein’s interactions with mammarenaviral NPs, which include that of LASV [122]. DDX3 was found to support viral growth as DDX3 knock-out (KO) cells were found to reduce replication of LASV and LCMV [122]. It has therefore been hypothesized that mammarenaviral NPs use DDX3 to sequester DDX3-interacting proteins (e.g., PKR, RIG-I, MDA5, and MAVS) that usually participate in IFN-I induction [122,123].

LASV Z protein has also recently been found to serve as another negative regulator of the cellular IFN-I pathway [18,19,21–24]. One of the mechanisms of how Z protein inhibits IFN-I expression is via its direct physical interaction with the eukaryotic translation initiation factor elF4E. Z binding to elF4E leads to a conformational change of the eIF4E’s 5ʹ cap binding site, thus reducing its affinity for cellular 5ʹ cap structures and reducing translation of elF4E-dependent cellular proteins, such as the IRF-7 transcription factor [24]. There is a positive regulatory feedback loop between IRF-7 and IFN-I during an antiviral immune response. However, as Z reduces the amount of IRF-7 expression, this positive feedback loop to activate IFN-I is disrupted [124–127]. Z protein has also been shown to antagonize the dsRNA-induced innate antiviral response of RIG-I by disrupting the complex formation between RIG-I and MAVS and thus inhibiting IFN-I induction [18,19]. It is noteworthy that a significant amount of natural sequence diversity in the Z gene suggests that natural viral gene variations may serve as a potential pathogenicity factor. For example, we have recently shown that natural sequence variations in the mammarenaviral Z proteins of human pathogenic LASV and LCMV serve as stronger inhibitors of the cellular innate immunity than those natural Z protein variants derived from mammarenaviruses that are either not known to cause diseases in humans or from naturally infected rodent reservoirs [18,19].

## Animal models for LF

Models for LF have been developed using small animals (e.g., mice and guinea pigs) and NHPs, however, only NHPs show clinical features of LF which mimick those of humans [for reviews, see 79, 128]. Briefly, wild-type, immunocompetent mice are naturally resistant to LASV infection and do not develop LF signs and symptoms, which limit their use in researching LF disease pathogenesis, therapeutic and vaccine developments. There are multiple NHP models for LASV infection, however, macaques are the most well studied and are considered the “gold standard”. Macaques develop a disease very similar to clinical cases of human LF, thus making them an excellent model for vaccine and antiviral evaluations, and pathogenesis studies [79]. Macaques exposed to LASV develop lethargy, anorexia, rash, fever, leukopenia, thrombocytopenia, elevated AST levels, and succumbed to the disease [129,130]. Like humans, viremia levels correlate with disease and survival outcome in LASV-infected macaques [129,130]. Despite these similarities, there are several important differences between NHP and human LASV infections. Hepatocellular necrosis is seen in human LASV infection, whereas NHPs only show focal areas of necrosis [96]. NHP models also show systemic and pulmonary arteritis, and elevated partial thromboplastin times (i.e., a test to measure how long to form a blood clot) which are not a common feature of human LF disease [131]. Along with some differences in disease presentation, efficacy studies in BSL-4 containment are extremely expensive. The use of NHPs in LF research, therefore, is hindered by the available numbers of the animal, high maintenance costs, and ethical concerns pertaining to their use. Due to these reasons, surrogate models of LF have been developed, including infection of small animals with Pichinde virus (PICV) [132,133] and LCMV, which have recently been reviewed elsewhere [79].

Recently, Safronetz and colleagues have successfully established a breeding colony of the wild-caught natural host of LASV, *M. natalensis* and used it to model LASV infection [52,134]. As expected, LASV infection of *M. natalensis* under the laboratory setting did not result in any significant changes in body mass during the 10-day pilot study, despite these animals exhibiting detectable levels of viral RNAs in their major organs. In a related study, Hoffmann and colleagues experimentally infected *M. natalensis* with the Morogoro virus (MORV), which is a nonpathogenic mammarenavirus that shares the same natural rodent reservoir as LASV, and another nonpathogenic mammarenavirus called Mobala virus (MOBV), which does not share the *M. natalensis* as a natural host [135]. Interestingly, the authors found that animals infected with MORV up to 2 weeks after birth developed persistent infection and were able to transmit the virus horizontally, whereas older animals (e.g., older than 2 weeks of age at the time of the infection) were able to rapidly clear the infection. On the contrary, MOBV was not able to establish persistent infection in young animals (i.e., neonates), which did not transmit the virus. It is therefore possible that mammarenavirus infections of *M. natalensis* is treated differently by the immune system of these rodents and should warrant further investigation. On that front, it is worth noting that Tang-Huau and colleagues have recently screened a large panel of commercially available rat and mouse antibodies against T cell receptors (CD3, CD4, CD8) and effector molecules (TNF-α and IFN-γ) for their potential cross reactivity with *M. natalensis* splenocytes, and found that the adaptive cellular immune responses by lymphocytes of *M. natalensis* to commonly used mitogens (e.g., phytohemagglutinin P, lipopolysaccharide and concanavalin A) are uniquely different from those of a laboratory strain of mice, such as C57BL/6 J mice [136]. This and similar future efforts are absolutely necessary in order to establish and optimize protocols to evaluate immune responses, such as lymphocyte proliferation and cytokine production in this wild-caught rodent model for the accurate evaluation of the unique immunological properties of these animals as reservoirs for mammarenaviruses and other equally important emerging and reemerging human pathogens (e.g., *Leishmania spp., Yersinia spp., and Borrelia spp*.).

## Current diagnostic, therapeutic and vaccine developments for LF

### Opportunities and challenges for LF diagnostic development and application

A challenge in West Africa in diagnosing LF patients is that it shares very similar initial clinical presentations with other febrile illnesses. LF is often diagnosed in patients only after anti-malarial and antibiotic treatments have failed to treat the disease, leading to a delay in the necessary LF patient isolation and treatment. Identifying the febrile illness source quickly enough to provide beneficial treatment requires a validated and rapid diagnostic tool. A definitive method of LF diagnosis is through a successful virus isolation; however, this is an impractical method in endemic areas since high biocontainment laboratory (i.e., BSL-4) is usually not available and the method is time consuming.

Although commercial polymerase chain reaction (PCR) and serology assays are available for LASV diagnosis, they are mainly used for research purpose only. Many international laboratories develop their own PCR, enzyme-linked immunosorbent assay (ELISA), immunoblot, and magnetic bead-based assays to detect LASV using published or unpublished protocols [137]. Reverse-transcription polymerase chain reaction (RT-PCR) has become commonly used because it can quickly detect the virus early in the course of the infection. Due to the relatively high sequence diversity of LASV clinical isolates geographically located throughout West Africa, RT-PCR-based assays can become an issue as even single nucleotide variations in the natural viral genomes can cause primer-template mismatches that have been shown to negatively impact assay sensitivity [138]. There are, however, strategies to solve this problem, including the use of multiplex panels to simultaneously detect different strains of LASV.

While PCR-based assays can be used as a sensitive and quick method for LASV diagnosis, rural healthcare units in LF endemic regions currently don’t have easy access to thermocyclers and/or the necessary molecular reagents, which render PCR-based methods for routine testing limited in its utility. Alternative methods for LF diagnosis include serological assays, such as immunofluorescence assay (IFA), ELISA, western blots, and multiplex bead assays. A traditional method for LASV serodiagnosis is an IFA that uses virus specific fluorescently tagged antibodies to bind to the virus in the sample and allow visualization. IFAs have mostly been replaced by ELISA to minimize time, labor, and biosafety requirements [138]. Other serological tests include ELISA that can be used to detect IgM and IgG antibodies against LASV that are naturally formed in LASV-infected individuals after a certain period post exposure to the virus [139]. Even though ELISA that is based on viral antigen and cellular antibody interactions is less specific than PCR-based assays, this method can allow for a greater level of flexibility in detecting diverse LASV clinical isolates [138]. Pan-ELISAs are designed to use polyclonal antibodies against several strains of LASV to allow for the detection of multiple clinical isolates of LASV in a single test [140]. Lateral flow assays have also been adapted to detect multiple strains of LASV at once and have been shown to have similar results to the Pan-ELISAs. Lateral flow assays are based on the same principle as ELISAs, that is dependent on an analyte in the sample to interact with reactive molecules which show a visual positive or negative result, but the lateral flow assays are paper-based, cheaper, and easier to use than ELISAs.

In addition to ELISAs and lateral flow assays, western blotting can also be used for LASV detection; however, it does not appear to be as commonly used in clinical settings as other methods. Briefly, western blotting is an analytical technique used to detect specific proteins in a sample. Proteins in the sample are separated by size and isoelectric charge during gel electrophoresis. Once the proteins are separated on the gel and are transferred onto a nitrocellulose membrane, they are made accessible to antibodies that bind specifically to the protein(s) of interest to allow for visualization via different chemoluminescence techniques. For example, a recombinant LASV NP protein can be used to detect anti-Lassa IgM antibodies present in the patient sera with a specificity of 90%-99.3%, depending on the patient’s samples [141]. Alternatively, magnetic bead-based assays, such as the Luminex MAGPIX platform, which uses analyte-specific beads covered in captured antibodies in combination with biotinylated detection antibodies to make an antibody-antigen “sandwich”. This antibody-antigen “sandwich” is then subjected to a dual-laser flow-based detection instrument which can detect the presence of the beads and the biotinylated antibodies to indicate a positive result. Magnetic bead-based assays have the advantage of being able to detect diverse clinical isolates of LASV because they can detect theoretically up to 50 discrete molecular targets in a single well [142]. MAGPIX developed by Satterly and colleagues had excellent reproducibility at a lower detection limit than ELISAs to detect LASV and Ebola virus (EBOV) antigens and the IgM antibodies against them [143]. Inactivated viruses, recombinant viruses, and virus-like particles (VLPs) have also been linked to magnetic beads to successfully detect immunoglobulins (or antibodies) in patients who have been infected with hemorrhagic viruses, making it a more convenient assay than those that use inactivated virus preparations [142]. Magnetic bead-based assays have technological requirements like PCR-based methods, and as such, this method is not practical for use in low income, resource poor and rural areas. However, efforts are being made to allow magnetic bead-based assays more accessible. For example, Adams et al. describe a self-contained magnetic bead assay format that can extract infectious disease biomarkers from complex biological samples and is designed for low resourced laboratory settings [144].

As LF symptoms are very similar to other endemic and febrile diseases in West Africa, having a method to perform differential diagnosis would be highly beneficial for making a correct disease diagnosis to start proper treatments as early as possible. Toward this end, RT-PCR 8-plex reaction assay can detect the expression of eight specific genes of different pathogens in a single reaction [145]. Additionally, oligonucleotide microarray platforms have been developed to differentiate related pathogens, or those with enough sequence divergence that would prevent the binding of the PCR primers to the DNA sequence of the pathogens. The number of potential genes detected per microarray far exceeds that of any other known technology, thus giving it the potential for highly multiplexed differential diagnosis of infectious diseases. Quan and colleagues developed a microarray platform (GreeneChipPm) for pathogen surveillance and discovery, which has viral oligonucleotide probes designed to represent at least three distinct genomic targets for every family or genus of viruses included in the assay [146]. GreeneChipPm was able to detect an infectious agent that was consistent with the diagnosis obtained from PCR or culturing technique using samples of patients with respiratory diseases, hemorrhagic fever, tuberculosis, and urinary tract infections. Currently, the diagnostics available for LASV outside of the laboratory do not take into consideration the genetic and geographical diversity of LASV lineages. The ability to differentiate these viruses in clinical diagnostics is desperately needed for an accurate diagnosis. However, differential diagnostics used in a laboratory setting often require instrumentation that is unavailable in low-resource areas. Future diagnostics would ideally be able to detect all known lineages of LASV with a high level of sensitivity and for use in a low-resource setting for on-site diagnosis of suspected LF cases.

## LF Therapeutics

### Ribavirin and favipiravir treatments

Treatment options for LF are often limited to supportive care (e.g., treating hypovolemia, electrolyte imbalances, and any concurrent bacterial infections as well as monitoring of coagulation factors) and an antiviral drug called ribavirin [5]. Ribavirin has been used for LF and other closely related viral hemorrhagic fevers with limited and varying degrees of clinical efficacy in humans [81,147,148]. According to the WHO, ribavirin has become a standard treatment for LF despite relying heavily on a single study by McCormick et al. [81], in which currently acknowledged standards were not used to confirm the clinical diagnosis of LASV in study participants [63]. For example, the convalescent plasma, which is an unproven intervention, was used as a comparison group to evaluate ribavirin efficacy. Also, evidence of ribavirin efficacy was only observed in a subset of patients with aspartate aminotransferase (AST) values > 150 U/lL while efficacy was not demonstrated in other patients.

Due to the lack of clinical evidence behind the recommended use of ribavirin for LF treatment, Eberhardt and colleagues conducted a systematic review and meta-analysis of all published and available data to assess clinical evidence of intravenous ribavirin efficacy [149]. Again, the authors found that international recommendations for ribavirin usage were primarily based on evidence from the McCormick clinical trial and a limited number of retrospective studies. They were also in agreement with the WHO that the McCormick clinical trial was not properly randomized and had other critical limitations in conducting, analyzing, and reporting experiments and data. Thus, in agreement with WHO, the authors concluded that international guidelines for use of ribavirin treatment of all LF patients lack substantiated evidence [149].

Oral administration of ribavirin, although less efficacious than intravenous ribavirin, has been recommended for postexposure prophylaxis (PEP) despite not having data supporting its use in humans, administration guidelines, or recommendations for duration of therapy [150,151]. Eberhardt and colleagues found that LF associated mortality is reduced for patients with elevated levels of AST when treated with ribavirin [149]. However, quite the opposite was found for ribavirin-treated LF patients who had non-elevated AST levels. LF patients with normal AST levels had higher fatal disease outcomes when treated with ribavirin, and this goes against the current treatment recommendations and medical practices in endemic regions which encourages the use of ribavirin in even mild cases. Although further analysis is needed, it appears that the liberal use of ribavirin could put some mild LF patients at an increased risk of death.

Several animal models have been performed to assess the effects of antivirals on LF infection. Mice are probably most widely used animal models as there are many different strains of mice to choose for these studies [152]. Ribavirin has been tested intravenously at 80 mg/kg per day in a chimeric mouse model called Ifnar^–/–B6^ mice. Ifnar^–/–B6^ mice are lethally irradiated IFN-I KO mice with transplanted bone marrow progenitor cells from wild-type mice, which provide them with a near complete mouse hematopoietic immunity and susceptibility to wild-type LASV infection [128]. Only ribavirin treatment that started on day 0 after LASV infection resulted in a prolonged survival time of the animals compared to the placebo group with no significant effect on viremia. Increased intravenous dosage of ribavirin to 160 mg/kg per day significantly prolonged survival duration and resulted in 20% of the animals surviving the infection. However, 160 mg/kg per day is significantly higher than what is recommended by WHO for human use. WHO currently recommends an intravenous ribavirin loading dose of 30 mg/kg, maximum of 2 grams, followed by 15 mg/kg, maximum of 1 gram, every 6 hours for four days [110,153]. At these specific concentrations for human use, ribavirin can cause hemolytic anemia, which is caused by a reduction in intracellular guanosine triphosphate (GTP) levels, and other severe complications [154], which can limit the biologically relevant doses for testing purpose in humans. Often the unintended clinical complications from ribavirin usage either alone or in combination with other therapies are severe enough to force a dose reduction or cessation of ribavirin treatment [155].

Ribavirin has also been tested in LASV-infected strain 13 guinea pigs, alongside ST-193, which is a small-molecule inhibitor of LASV entry [155]. Strain 13 guinea pigs are an immunologically competent inbred strain that, when infected with LASV, can show many of the same clinical features of LF as humans [70,155,156]. Strain 13 guinea pigs were injected by intraperitoneal (i.p.) route with either ribavirin or ST-193 once before LASV infection at 1,000 plaque forming units (pfu) of LASV-Josiah strain and then were treated daily for 14 days. Morbidity scores for mock and ribavirin-treated animals were significantly higher than those of the ST-193-treated group [155]. While all control (mock infection with buffer saline) and all ribavirin-treated animals died between 13–25 days post-infection, 60% of ST-193-treated animals resolved the infection by day 19 (albeit these animals exhibited weight loss and fevers initially). Although Strain 13 guinea pigs are a useful model and are uniformly susceptible to lethal infection (with only 2 or more PFU of the LASV Josiah strain), they lack the genetic diversity to mimic human populations. In contrast, outbred Hartley guinea pigs are genetically diverse and are more widely available than Strain 13 guinea pigs. However, outbred Hartley guinea pigs do not exhibit consistent morbidity and lethality without viral adaption of LASV strains via serial passaging [157]. In contrast, infection of outbred Hartley guinea pigs with the LASV isolate (LF2384), which was isolated from a serum sample of a fatal LF human case during the 2012 Sierra Leone outbreak, showed uniform and lethal infection, which could be useful for future vaccine and antiviral studies [126]. Similarly, infection of STAT1 KO mice with this strain of LASV produced similar levels of uniform and lethal infection [9].

Besides studies with mice and guinea pigs, there are other studies examining ribavirin usage in treating LASV infection in NHPs [134,158,159]. One of the first studies was carried out in 1984 by the Jahrling group. In this study, cynomolgus macaques were treated with the LASV-infected monkey serum, ribavirin, or both. Monkeys that were treated early (0–4 days post infection with LASV) in all three treatment groups survived. If treatments were initiated after 7 days post infection, only 16% of serum-only treated animal survived and 50% of ribavirin-only treated animals survived. However, 100% of the combination (serum and ribavirin)-treated animals survived, even when treatment started 10 days after infection with LASV [158]. In another NHP study, Lingas and colleagues studied the viral dynamics of NHPs treated with favipiravir, a broad-spectrum viral polymerase inhibitor that was initially designed for influenza and has been shown to dampen LASV viremia in animal studies [110,139], or ribavirin, and found that ribavirin had an antiviral efficacy *in vivo* [134]. Animals that were treated with ribavirin (30 mg/kg) had a median survival of 20.5 days while untreated animals had a median survival of 10 days. The authors also found from their modeling of drug efficacy for human dosing regimens, based on WHO’s guidelines for intravenous ribavirin dosage, that 1,000 mg every 6 hours would reduce LASV infectivity by approximately 80% [134]. However, there were limitations to this study. The modeling assumed LASV was not multicompartmental (i.e., having multiple biological effects) because there are currently no modeling techniques to take this into account. The authors did not consider the adaptive immune response to viral infection, but they pointed out that viral dynamics, after reaching a maximum level, showed a slow decline, suggesting that adaptive immune response to control the infection was limited in scope [134]. Overall, they concluded that ribavirin could be helpful in reducing the proportion of infectious virus at dosages relevant in humans.

Other studies have found that a combination treatment of favipiravir and ribavirin has a synergistic effect *in vitro* which lowers viremia levels and viral loads in the visceral organs of Ifnar^–/–B6^ mice infected with LASV. An increase in survival rate and time was seen for animals treated with a combined suboptimal dose of each of favipiravir and ribavirin [110]. Besides genetically engineered mice (e.g., IFN-I KO), guinea pigs have also been used to compare the efficacy of ribavirin and favipiravir combination treatment against a lethal viral challenge [157]. At two different dosages (150 mg/kg and 300 mg/kg), favipiravir-treated animals had statistically significant higher survival rates than the placebo and ribavirin only treated animals [157]. The animals treated with 150 mg/kg per day experienced weight loss between 2% and 16%, developed fevers, but survived the infection, while the animals treated with 300 mg/kg per day were fully protected. Only two animals in the 300 mg/kg treatment group developed fevers and no animals lost weight. To put the dosages used in this study into a human perspective, 300 mg/kg in a guinea pig is equivalent to 65 mg/kg in human, which is lower than the dose used for treatments during the 2014 Ebola outbreak [157]. Interestingly, the animals that were treated with ribavirin appeared relatively normal for much of the duration of the experiment, however, almost immediately after the cessation of treatment the animals developed disease signs and symptoms and ultimately succumbed to the infection [157]. The antiviral efficacy of favipiravir against LASV infection has also been assessed in cynomolgus macaques [160]. Treatment that started at four days post infection at 300 mg/kg daily effectively protected all four macaques in the treatment group from lethal LASV infection, unlike the control placebo group, all of whom succumbed to viral infection. Taken together, the lack of efficacy data for ribavirin usage in treating LF besides those reported in a single study done in humans [81] and international treatment guidelines for treating LASV infection that are based on it, strongly suggests that more studies need to be done in human clinical trials in order to assess the efficacy of ribavirin treatment in LASV-infected human patients in terms of its therapeutic effects against multiple LASV strains and lineages, different stages of the disease, different administration routes, and dosages.

### Other experimental drugs and strategies

Other researchers have been looking into repurposing combinations of already FDA-approved oral drugs to treat LASV (and other viruses) as a rapidly deployable defense in future virus outbreaks [161,162]. *In vitro* analysis showed that arbitol (a membrane fusion inhibitor) combined with aripiprazole (a cellular micropinocytosis inhibitor) or combined with sertraline (a membrane fusion inhibitor) can synergistically inhibit LASV or JUNV pseudovirus infection [160]. The same group of investigators also showed that arbitol, sertraline, and niclosamide were found to suppress LASV infection in cell culture. Investigating these already available drugs may be beneficial because most current drug countermeasures for acute viral infections are single agents. However, most successful antiviral therapies, especially for chronic viral infections, are based on multiple drug combinations. Many of these already available drugs can target different stages of the virus life cycle, or the host factors required for viral replication, and thus are able to reduce the emergence of drug-resistant viral strains [161,163]

Small interfering RNAs (siRNAs) have also been shown to inhibit LASV replication in *in vitro* studies [164]. Treatment of cells infected with different LASV isolates with siRNAs targeting the conserved LASV genomic RNA terminal sequences upstream of the viral NP and L genes has demonstrated antiviral activity by reducing reporter gene expression from the LASV replicon and LASV mRNA expression plasmids. Both NP and L siRNAs inhibited replication of LASV up to 1 log unit with no negative effect on cellular viability [164]. A downside of siRNA therapeutics is that siRNA must perfectly base pair with the targeted viral sequence, which can be problematic when considering the significant sequence diversity in LASV isolates [139,164].

Since the late 1970s, antibodies have been investigated *in vitro* and in clinical settings to treat LF [165,166]. Convalescent plasma has shown some promising results when given to patients early in the disease course. Human monoclonal antibodies (humAbs) derived from B cells of convalescent donors have also been studied in outbred Hartley guinea pig and cynomolgus macaque models of LF [167]. Human mAbs that cross-reacted with the glycoproteins of all four clades of LASV were found *in vitro* to bind LASV GP and to prevent binding of GP to its cellular receptor α-dystroglycan as well as to prevent envelope fusion with the host-cell membrane. Using a combination of humAbs, Mire and colleagues showed that they could rescue 100% of LASV-infected macaques who started treatment up to 8 days after virus challenge [168]. Therefore, humAbs have a significant potential as a LF treatment modality in humans and therefore warrant further investigations.

### LF vaccines

Although there are currently no FDA-approved vaccines for LF, there are many candidate vaccines in preclinical development that have produced variable but encouraging results (Table 1). In 2017, the WHO released their Target Product Profile (TPP) for LF vaccines and emphasized that a prophylactic vaccine has the highest priority [169]. It was stated that the optimal candidates for an LF vaccine should meet WHO-acceptable safety/reactogenicity, be single-dose, be greater than or equal to 70% efficacy in preventing infection or disease caused by the LASV lineages I–IV, and be long lasting (greater than or equal to 5 years) [169, 8].

**Table 1.** Lassa vaccine candidates and platforms used in preclinical developments

Vaccine Candidate	Animal Model	Antigen or Virus Strain	Dose Number	Dose	Days to Challenge	Survival Rate (%)	Test Parameter	Date	Reference
Recombinant vesicular stomatitis virus	Guinea pig – strain 13	GPC and NP - Josiah	1	10^6 PFU	28	100	Antibody	2015	[157]
	Cynomolgus monkey	GPC – Josiah	1	2–6^7 PFU	28	100	Antibody and T cell IFN-gamma	2005,2015	[157,182]
DNA	Guinea pig – strain 13	GPC – Josiah	3	100 µg	63	100	Antibody and T cell IFN-gamma	2013	[191]
Recombinant yellow fever 17D	Guinea pig – strain 13	GPC – AV	1	10^5 PFU	21	80	Antibody	2006	[193]
	Guinea pig – strain 13	GP1 and GP3 – Josiah	2	5^6 PFU	44	83	Antibody	2011	[194]
Recombinant Mopeia/Lassa Fever (ML29)	Guinea pig – strain 13	GPC and NP – Josiah	1	10^3 PFU	30	100	Antibody	2005	[178]
	Marmoset	GPC and NP – Josiah	1	10^3 PFU	30	100	Antibody and T cell IFN-gamma	2008	[105]
Vaccinia (NYBH) vectored virus	Rhesus and cynomolgus monkeys	GP1, GP2, GPC, NP – Josiah	1,2	10^9 PFU	62–488	90-GPC/NP	Antibody and T cell IFN-gamma	1989	[192]
Nanocarrier rGP1 encapsulated into polymersomes	C57BL/6 mice	GP1 – unknown	2	10 µg	14 and 28	0 – all animals sacrificed at 28 days, no survival data	Antibody/CD4 T cell/B cell	2017	[42]
Venezuelan equine encephalitis virus replicon	Guinea pig – strain 13	GPC and NP – Josiah	1	1^5 PFU	21	80		2006	[193]
Chimpanzee adenovirus – ChAdOx1-Lassa-GP	Hartley guinea pig	GP – Josiah	2				Antibody/CD4 T cell		[170]
	Mice and guinea Pigs	GPC-Josiah LASV	1	1^8 IU or 3^8 IU	28 and 56	100	Antibody/CD4 T cell	2021	[177]
Chimpanzee adenovirus -Ad5 (E1 and E2b-deleted)	Hartley guinea Pigs	GPC and NP - Josiah	2	1^10 IU	40 and 56	100	Antibody	2019	[127]
Lassa virus-like particles	BALB/c mice	Z, GPC, and NP – Josiah	2	10 µg	N/A	0 – Challenge virus not assessed	Antibody/CD4 T cell	2010	[69]
Inactivated LASV	Rhesus monkeys	Inactivated LASV – unknown	1	1^4 PFU	108	0	Antibody	1992	[196]
Measles Virus (MV) backbone	Cynomolgus monkeys	LASV Josiah GPC and NP	1	2^6 TCID50	37	100	Antibody/CD4 T cell	2021	[174]
Alphavirus	CBA/J mice	VLPs – GPC genes of clades I and IV, LASV/NIG/LP and LASV/Josiah.	21	Challenge virus not Assessed			Antibody/CD4 T cell	2018	[195]

*PFU: Plaque forming units, IU: Infectious units.

Most vaccine strategies and platforms have so far been investigated and developed using the Josiah LASV GP antigen and have provided protection during homologous challenge with the same strain of the virus, but not against heterologous virus challenge [13,100]. Since cellular immunity is favored over humoral immunity in clearing LASV infection, GP and NP antigens have been chosen for vaccine formulations in especially viral vector-based and live-attenuated LF vaccines, because they can induce strong CD4 and CD8 T-cell responses and can persist for years [106,170].

The first phase I trial to evaluate a LF vaccine candidate’s safety and immunogenicity, called INO-4500, was completed in October 2021, however no results have been posted at the time this article was published. The INO-4500 is a DNA-based vaccine candidate that is based on the backbone of the measles virus (MV) Schwarz strain and expresses the LASV GP The INO-4500 trial was a prospective, interventional, observer-blinded clinical trial with 52 study participants. The recombinant MV vaccine platform can express antigens from various pathogens and is proven to be safe, stable, immunogenic, and effective in several studies [171,172]. A Schwarz MV vaccine platform has also been used to develop a candidate vaccine for LF. This vaccine expresses the Josiah strain LASV GPC, Z, and/or NP. Compared to other formulations (e.g., GPC and Z or GPC alone which did not provide as much protection), the MV-based vaccine expressing the LASV GPC and NP provided the most protection in cynomolgus monkeys against LASV (Josiah strain) challenge. All monkeys vaccinated with the MV-NP vaccine remained healthy, and only had a slight increase in clinical scores due to small increases in body temperature. The vaccine also appeared to provide sterilizing immunity as there was no replicating virus found in any of the treated animals upon a virus challenge [173]. The MV-NP vaccine had moved into phase I human clinical trials, which were completed on 15 January 2021. However, results have not yet been disclosed at the time of this article’s publication [43]. It therefore remains to be seen whether and what levels of protection afforded by this MV-NP vaccine are in vaccinated individuals. The same group of investigators has recently reported results of a follow-up study of their MV-based vaccine expressing the LASV Josiah GPC and NP against heterologous strains of LASV in cynomolgus monkeys to show that the vaccine was protective against lineage II or lineage VII of LASV when the animals were challenged one month after vaccination. The investigators also found that even a single dose of the MV-NP vaccine was sufficient to protect monkeys against the homologous Josiah strain challenge one year later [174].

Recent developments of adenovirus vector-based platforms, such as human Ad5 and Ad35 and chimpanzee adenoviruses (ChAd3, ChAdOx1, ChAd63), as vaccine vectors to express immunogenic proteins of influenza and other important pathogens, including LASV, have progressed at a fast pace with some of these viral vectored vaccines entering different human clinical trials [for reviews, see 175, 176,]. An Ad5 (E1 and E2b-deleted) vector-based vaccine expressing the LASV NP or GPC protein was found to protect guinea pigs against lethal LASV challenge in a prime-and-boost vaccination strategy [126]. Additionally, a single vaccinated dose of the ChAdOx1 vector-based vaccine expressing the Josiah LASV GPC has been shown to induce robust T-cell and antibody responses in mice and can protect guinea pigs against morbidity and mortality following lethal challenge of the vaccinated animal with a guinea pig-adapted Josiah LASV strain. A prime-and-boost vaccination of this vaccine has also been shown to significantly enhance LASV antigen-specific antibody titers and clear LASV from the tissues of the virus-challenged animals [177]. Therefore, adenovirus vectored LASV vaccines have shown some encouraging results in guinea pig models that warrant additional testing in the gold-standard non-human primate LF model.

Other promising vaccine candidates include the live attenuated mammarenavirus ML29, recombinant vesicular stomatitis virus (VSV), and vaccinia-vectored vaccine platforms [for a review, see [100]]. As genetically related mammarenaviruses can undergo reassortment by exchanging their genomic RNA segments during coinfection, ML29 was created from reassortment of the genomic RNA segments of Mopeia virus (MOPV) and LASV (Josiah strain) by which the L segment of MOPV and S segment of LASV are reassorted (or packaged) into a single virion. Carrion and colleagues first tested this ML29 vaccine in the inbred strain 13 guinea pigs [178]. Vaccination of guinea pigs with a single subcutaneous (s.c.) dose of ML29 30 days before challenge with 10^3^ PFU of LASV provided complete protection against LASV infection.

It has been demonstrated that a single s.c. inoculation of LASV in marmosets can result in a systemic disease with fatal outcomes [179]. Additionally, similar histological features were observed in these animals as those seen in LASV-infected humans, which made this an excellent model for understanding LF disease pathogenesis and for vaccine efficacy studies. ML29 had also been shown to completely protect common marmosets against LASV infection (at 10^3^ PFU) when a single s.c. dose of 10^3^ PFU of the ML29 vaccine was used [105]. Vaccinated marmosets had neither clinical symptoms nor changes from pre-challenged values of blood chemistry and hematological data. All vaccinated marmosets survived the observational period of 35 days after LASV challenge. In contrast, the control animals, which were immunized with a diluted condition medium of Vero E6 cells and then challenged with 10^3^ PFU LASV, showed disease symptoms, such as reduced platelet numbers, elevated liver enzymes, decreased plasma albumin levels, and none of these animals survived. Immunization of marmosets with ML29 induced specific cell-mediated T cell responses that seemed to confer complete protection, as evidenced by the lack of histological alterations and by the clearance of blood and tissues of infectious LASV.

A recent experiment of ML29-infected Vero E6 cells at a low multiplicity of infection (MOI) or persistently infected cells, initially at a high MOI, showed that the viral L segment-derived and truncated viral RNA species can be readily detected in these cells [180]. The abundance of truncated ML29 RNA species has been shown to contribute to a high degree of attenuation and immunogenicity of the ML29 vaccine when it was tested in the immunodeficient STAT-1 KO mice as well as in immunocompetent mouse and guinea pig models, although the exact mechanisms behind it are unclear [180]. The authors of this study have suggested that a blended formulation of LASV candidate vaccine with truncated, defective interfering viral particles (DIP) can be considered in vaccination to induce broadly cross-reactive antibodies against LASV strains of all seven lineages of LASV [180]. Although data on ML29 and its attenuated vaccine formulations look promising, their utility as vaccines against LF needs to take into consideration the fact that West Africa has a high population of people living with altered immune statuses (e.g., immunosuppression due partly to either HIV-1 or other pathogen infections). From a preliminary study done in Nigeria, it is estimated that LASV seroprevalence is three times higher in HIV-positive individuals than in HIV-negative individuals, thus indicating that both LASV and HIV-1 infections co-exist in Nigeria and target the same human population [181]. The risk of adverse effects of the recombinant MV-based LASV vaccine platform or ML29 with DIP blended or not needs to be further studied in HIV populations before being considered as a population-based vaccination strategy.

Recombinant VSV (rVSV) is another viral platform that has shown some promising results as advanced vaccine candidates [for a review on this and other viral vectored vaccines, see [175]]. VSV is perceived to be advantageous due to its high level of immunogenicity and the lack of preexisting immunity to this viral vector in human populations [182]. rVSV is a safe vaccine platform because it is engineered to lack the glycoprotein (G) gene, which is a known major viral pathogenic factor; and in its place, either the LASV GP or other gene(s) of pathogens like EBOV and Marburg virus (MARV) is inserted [183]. This virus vectored platform was used to express the glycoproteins of EBOV and MARV for testing in NHPs that showed promising results; and some of these candidate vaccines, such as the Ervebo EBOV vaccine, have been evaluated in clinical trials and approved for human use by the FDA [183]. Additionally, the rVSV-LASV vaccine, called VSVΔG/LVGPC, that expresses the glycoprotein of LASV Josiah strain has been used to vaccinate cynomolgus macaques [182]. The macaques received a single intramuscular (i.m.) dose at 2 × 10^7^ PFU of the vaccine and were challenged 28 days post-vaccination with 10^4^ PFU of LASV. Whereas the control animals, which were immunized with the same vaccine platform except with the Zaire ebolavirus (ZEBOV) GP instead of LASV GP, succumbed to LASV challenge and had pathological changes consistent with other LASV NHP studies, those NHPs vaccinated with VSVΔG/LVGPC were fully protected against LASV challenge. Furthermore, rVSV vector replication and shedding were undetectable in the vaccinated animals, highlighting its safety profile. Furthermore, VSVΔG/LVGPC vaccination studies in outbred Hartley guinea pigs and NHPs have shown complete protection against heterologous LASV challenge as well as homologous LASV challenge [190]. It is noteworthy that the rVSV approach is the first vaccine platform to demonstrate cross-clade protection against all major lineages of LASV [184]. The quadrivalent VesiculoVa consists of recombinant vesicular stomatitis virus (rVSV) vectors expressing filovirus glycoproteins as well as a rVSV vector expressing the glycoprotein of the lineage IV strain of LASV. When cynomolgous macaques were vaccinated via a prime-and-boost strategy with this quadrivalent vaccine, they were found to be protected against not only three filoviruses (Ebola, Sudan, and Marburg) but also a heterologous linage II LASV strain. However, rVSV is a replication competent virus that may induce some unintended clinical symptoms in vaccinated humans, and as such, the widespread use of the VSVΔG/LVGPC needs to be further evaluated.

Another promising virus-vectored vaccine platform currently being developed is the recombinant vaccinia viruses (VACs) expressing LASV NP and GPC [104]. Using a New York Board of Health (NYBH) strain of vaccinia virus, VAC-LASV candidate vaccines were created to express different portions of the LASV GPC and NP proteins as antigens and were used to immunize NHPs to study the protective efficacy of individual LASV antigens [104]. A full-length LASV GPC as an antigen was needed to provide a full level of protection of the VAC-LASV vaccinated and LASV challenged animals. Animals vaccinated with either VAC-based vaccines expressing the full-length GPC or the full-length S-segment that expresses the GPC and NP showed significantly diminished LASV titers in vaccinated animals as compared to unvaccinated animals when they were challenged with LASV [104]. Despite these promising results, using a VAC-based vaccine platform in Africa with a population with a high prevalence of HIV-1 positive individuals who are immunosuppressive needs further investigations and additional considerations.

## Concluding remarks

Based on the current estimate, 37.7 million people who are living in Africa are at risk of contracting LASV, yet no effective therapeutics or vaccines are currently available against this deadly human pathogen. LASV has high genetic diversity across different lineages and similar disease symptoms as other febrile illnesses, which make a correct disease diagnosis challenging. Although there are some diagnostics available for LASV, laboratory-developed protocols are not usually made for clinical settings, especially in resource-poor areas where LF is endemic. Because diagnostics are essential for surveillance and prevention of LASV outbreaks, efforts to advance existing diagnostic platforms toward clinical laboratory validation and meeting regulatory approvals necessary for clinical application are desperately needed. Future diagnostics technology must also be simple, inexpensive, and sensitive enough to distinguish the genetic and geographical diversity of LASV clinical isolates. Supportive care and ribavirin antiviral are the only approved therapeutics available for LF treatment. Ribavirin has varying degrees of efficacy depending on disease severity and time of administration. Often by the time LF is diagnosed, it is already too late for ribavirin to offer any clinical benefits. Ribavirin is teratogenic and therefore is contraindicated in pregnant women who are infected with LASV. Aside from ribavirin, there are other LF therapeutics in development, but none has advanced into the clinical setting. As for vaccines, many candidates are in preclinical development with very few in clinical testing stages. In the meantime, continuing efforts to understand the basic biology of LASV and its immunological and pathological impacts on the infected individuals will offer important insights into the molecular mechanisms of virus virulence, pathogenicity, and disease pathogenesis. Additional efforts to identify and characterize circulating LASVs and mitigating efforts to control the rodent vector populations that harbor these deadly viruses, and their geographical distributions are essential until an effective treatment or prevention strategy is available [43,185–189].

## Data Availability

All figures and table included in this article have been deposited in a recognized data repository (Figshare.com) with a digital object identifier (10.6084/m9.figshare.14350274).
